# Long-Lasting Changes of Repeated Anodal Transcranial Direct Current Stimulation in Decreasing Chronic Pain in Patients with Multiple Sclerosis: A Case Series

**DOI:** 10.3390/brainsci16070767

**Published:** 2026-07-22

**Authors:** Renming Liu, Shapour Jaberzadeh, Mona Malekahmad, Murray Taverner, Jamie Young, Maryam Zoghi

**Affiliations:** 1 Monash Neuromodulation Research Unit, Department of Physiotherapy, School of Primary and Allied Health Care, Monash University, Melbourne, VIC 3199, Australia; 2 Department Perioperative Medicine, Nursing and Health Sciences, Monash University, Clayton, VIC 3168, Australia; 3 Frankston Pain Management, Pain Medicine Unit, Peninsula University Hospital, Melbourne, VIC 3199, Australia; 4 Department of Critical Care, The University of Melbourne, Melbourne, VIC 3010, Australia; 5 Department of Anesthesia, Perioperative and Pain Medicine, Peter MacCallum Cancer Centre, 305 Grattan St, Melbourne, VIC 3000, Australia; 6 Discipline of Physiotherapy, Institute of Health and Wellbeing, Federation University, Melbourne, VIC 3353, Australia

**Keywords:** case series, corticospinal excitability, multiple sclerosis, transcranial direct current stimulation, transcranial magnetic stimulation

## Abstract

**Highlights:**

**What are the main findings?**

- Repeated anodal tDCS over the primary motor cortex was associated with reductions in pain intensity in six individuals with MS-related chronic pain.
- Anxiety often decreased alongside pain, while TMS-derived neurophysiological measures showed marked inter-individual variability, with no consistent relationship between pain reduction and corticospinal excitability changes.

**What are the implications of the main findings?**

- Individual longitudinal assessment may be important when evaluating neuromodulation responses in MS-related chronic pain, as group-level outcomes may obscure clinically meaningful response trajectories.
- These findings support future controlled studies investigating repeated-block or maintenance anodal tDCS protocols and potential moderators of response, including affective symptoms and baseline neurophysiological profiles.

**Abstract:**

**Background:** Pain affects 50–75% of individuals with multiple sclerosis (MS) and is more common than in the general population. Chronic pain is difficult to manage and may involve motor cortical and sensorimotor network alterations. Anodal transcranial direct current stimulation (tDCS) over the primary motor cortex (M1) has been explored as a neuromodulatory approach, although its effects and mechanisms remain unclear. **Objective:** This case series describes longitudinal changes in pain and anxiety following repeated anodal tDCS over left M1 in six individuals with MS-related chronic pain and descriptively reports concurrent changes in transcranial magnetic stimulation (TMS)-derived neurophysiological measures. **Methods:** Six adults with MS and chronic pain (≥4/10) received 1 mA anodal tDCS over M1 (10 min stimulation, 25 min interval, 10 min stimulation) in two 5-day blocks separated by 2 weeks (10 sessions total), with usual care maintained. Pain and anxiety (0–10) were assessed before and after sessions and at follow-up. TMS assessed corticospinal excitability and intracortical inhibition and facilitation at baseline, post blocks, and at follow-up. **Results:** Marked inter-individual variability was observed in clinical and neurophysiological trajectories following repeated anodal tDCS. Response patterns included immediate improvement after stimulation blocks, delayed improvement after the second block, and transient rebound during the inter-block interval. TMS-derived measures showed heterogeneous changes, with no consistent correspondence with clinical outcomes. **Conclusions:** This case series describes variable changes in pain, anxiety, and neurophysiological measures following repeated anodal tDCS over M1 in MS-related chronic pain. The findings are observational and highlight inter-individual variability, supporting further controlled studies.

## 1. Introduction

Pain is a highly prevalent and disabling non-motor symptom of multiple sclerosis (MS), affecting approximately 50–75% of individuals across the disease course [1]. MS-related chronic pain is clinically heterogeneous and multifactorial, arising from central nervous system pathology, secondary musculoskeletal dysfunction, and maladaptive neuroplastic changes [2]. Patients commonly report burning, electric shock-like, or stabbing pain, frequently accompanied by fatigue, sleep disturbance, anxiety, reduced mobility, and impaired daily functioning [3,4,5]. Accordingly, chronic pain in MS is increasingly recognised as a multidimensional condition with major consequences for quality of life and functional independence.

The neurobiological mechanisms underlying MS-related chronic pain remain incompletely defined. Demyelination, neuroinflammation, and axonal injury disrupt communication across sensory, motor, and affective networks, leading to widespread alterations in neural processing [6,7]. Although traditionally attributed to somatosensory pathway dysfunction, emerging evidence indicates that motor network abnormalities may also contribute to pain persistence [8,9]. In support of this, transcranial magnetic stimulation (TMS) studies have demonstrated altered corticospinal excitability (CSE) and intracortical inhibitory function in MS, with reduced CSE associated with greater pain and fatigue severity [10]. Collectively, these findings suggest that MS-related pain reflects distributed sensorimotor network dysfunction rather than isolated sensory pathway impairment.

TMS provides a non-invasive method for probing corticospinal and intracortical circuit physiology. Single- and paired-pulse paradigms enable quantification of CSE and intracortical inhibitory and facilitatory processes. Short-interval intracortical inhibition (SICI), long-interval intracortical inhibition (LICI), and intracortical facilitation (ICF) are widely used as indirect indices of cortical inhibitory and excitatory circuit function, reflecting, at least in part, processes related to GABAergic and glutamatergic neurotransmission [11]. Alterations in the balance between intracortical inhibition and facilitation have been reported across chronic pain conditions and are thought to contribute to maladaptive plasticity, impaired sensorimotor integration, and persistent pain states [12]. More recent neuromodulation research has conceptualised cortical excitability as a state-sensitive physiological measure that may help characterise within-subject neurophysiological change, while also recognising that stimulation responsiveness may depend on baseline physiological state, network responsiveness, and inter-individual variability in plasticity mechanisms [13,14]. In the present study, TMS-derived measures were therefore included as exploratory physiological descriptors rather than definitive biomarkers of clinical response.

Non-invasive brain stimulation has emerged as a promising adjunctive strategy for chronic pain management [15]. Among available techniques, anodal transcranial direct current stimulation (tDCS) targeting the primary motor cortex (M1) has received particular attention due to its ability to modulate cortical excitability and influence distributed pain-processing networks [16,17].

Contemporary neuroimaging and neurophysiological evidence position M1 as a key hub within sensorimotor and pain-modulatory networks rather than a purely motor structure [18,19]. Modulation of M1 may therefore influence pain processing through thalamocortical interactions, sensorimotor integration, and engagement of descending inhibitory pathways [20]. Prior studies have reported analgesic effects of anodal tDCS in MS populations [21]. However, most studies have focused on clinical outcomes, with limited understanding of underlying neurophysiological mechanisms or longitudinal response dynamics.

MS is characterised by marked inter-individual variability, including differences in lesion burden, disease duration, pain phenotype, medication exposure, fatigue severity, affective symptoms, and baseline cortical excitability. Such heterogeneity may substantially influence responsiveness to neuromodulation.

Accordingly, group-level analyses may obscure clinically meaningful individual response trajectories. In line with CARE (Case Report) reporting principles, an exploratory case series design was selected to enable detailed within-subject longitudinal characterisation of clinical and neurophysiological changes, with emphasis on hypothesis generation and mechanistic exploration rather than treatment efficacy estimation [22].

This exploratory case series investigated the effects of repeated anodal tDCS in six individuals with MS-related chronic pain. The primary aim was to characterise individual longitudinal trajectories of pain and anxiety following repeated stimulation. The secondary aim was to descriptively explore whether changes in clinical outcomes over time were accompanied by corresponding changes in TMS-derived measures at the individual level. By integrating clinical and neurophysiological measures, this study provides descriptive longitudinal observations to inform future mechanistic research on motor cortex-targeted neuromodulation in MS-related chronic pain.

## 2. Methods

### 2.1. Study Design and Reporting Framework

This study was a prospective, exploratory descriptive case series examining the clinical and neurophysiological effects of repeated anodal tDCS in individuals with MS-related chronic pain.

The study was conducted in accordance with the Declaration of Helsinki and approved by the Monash University and La Trobe University Human Ethics Committee. Written informed consent was obtained from all participants prior to enrolment.

As an exploratory case series, no formal sample size calculation was performed. Six participants were included to enable detailed within-subject longitudinal characterisation rather than between-group inference.

### 2.2. Participants and Recruitment

Participants were recruited consecutively between January 2023 and December 2025 through referrals from neurologists and pain specialists. Eligible participants were adults with a neurologist-confirmed diagnosis of MS and chronic pain persisting for at least three months. Pain phenotypes included burning pain, pins-and-needles sensations, electric shock-like pain, stabbing pain, dysesthesia, and allodynia. Pain intensity was assessed using 10 cm visual analogue scales (VAS), where 0 indicated no symptoms, and 10 indicated the worst imaginable intensity.

Participants continued their usual medical care throughout the study, including analgesic, antispasticity, antidepressant, anticonvulsant, and disease-modifying therapies. Medication use was documented at baseline and monitored throughout the intervention period to support interpretation of clinical and neurophysiological outcomes.

Exclusion criteria included contraindications to tDCS or TMS, including epilepsy or unexplained seizures, implanted metallic or electronic devices, unstable neurological or psychiatric conditions, pregnancy, or any condition deemed to increase stimulation-related risk.

### 2.3. Intervention: Anodal tDCS Protocol

Active anodal tDCS was delivered using saline-soaked sponge electrodes at 1 mA intensity. Each session consisted of two 10 min stimulation periods separated by a 25 min interval (total stimulation time: 20 min).

The anode (5 × 7 cm) was placed over the left M1, and the cathode (3 × 4 cm) over the contralateral supraorbital region. Electrode placement, impedance, and contact quality were verified before each session to ensure stimulation fidelity.

Participants received two treatment blocks, each comprising five consecutive daily sessions, separated by a two-week interval (total: 10 sessions). This repeated-block design was used to explore potential cumulative or reinforcement-related neuromodulatory effects. The protocol was informed by evidence that repeated anodal tDCS is spacing-dependent, with two 10 min stimulation periods separated by a 25 min interval associated with greater and more sustained CSE changes than shorter intervals in previous neurophysiological studies. This schedule was also consistent with a previous trial in MS-related chronic pain. However, the present exploratory study was not designed to determine dose–response relationships or identify the optimal stimulation schedule. Five daily sessions were delivered in each block, with a second block after a 2-week stimulation-free interval to descriptively examine symptom trajectories during the interval and after re-exposure [23].

**Figure 1:**
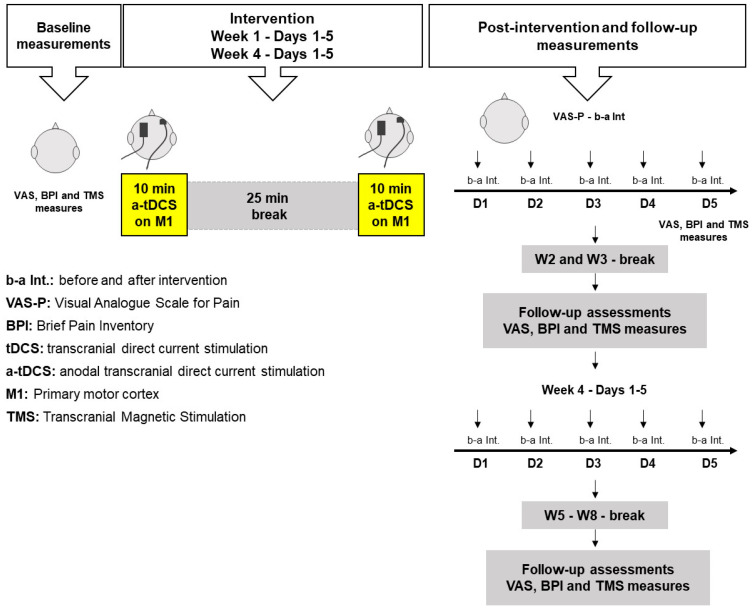
Study protocol. Participants completed baseline assessments before receiving two blocks of anodal transcranial direct current stimulation (anodal-tDCS) in Week 1 and Week 4. Each intervention block consisted of five consecutive daily sessions. On each intervention day, anodal-tDCS was applied over the M1 for 10 min, followed by a 25 min break and a second 10 min stimulation period. Pain and Anxiety Visual Analogue Scale (VAS) were collected before and after each daily intervention. TMS measures were collected at baseline, after each intervention block, and during follow-up assessments. Weeks 2–3 and Weeks 5–8 were break/follow-up periods without intervention. Arrows indicate the sequence and timing of the study procedures and assessments.

Participants were monitored during stimulation and systematically assessed for adverse effects, including scalp discomfort, tingling, itching, headache, dizziness, and fatigue. All adverse events, missed sessions, and deviations from protocol were recorded (Figure 1).

### 2.4. Clinical Outcome Measures

The primary clinical outcome was pain intensity measured using a 10 cm VAS. Secondary outcomes included anxiety intensity measured using an identical VAS scale, selected due to the high comorbidity of affective symptoms in chronic pain populations. The brief pain inventory (BPI) was also collected to provide additional descriptive information on pain severity and pain-related interference.

Assessments were conducted longitudinally at baseline, after Block 1, during the inter-block interval, after Block 2, and at 28-day follow-up. Particular emphasis was placed on within-subject response features, including immediate response, delayed response, symptom rebound during treatment gaps, and durability of effect.

All outcomes were analysed descriptively at the individual level.

### 2.5. Neurophysiological Outcome Measures (TMS and EMG)

TMS was used to assess CSE and intracortical inhibitory and facilitatory circuits.

Participants were seated comfortably with the tested upper limb supported to minimise background EMG activity. Surface electromyography (EMG) was recorded from the first dorsal interosseous (FDI) muscle using Ag/AgCl electrodes in a belly–tendon montage (inter-electrode distance around 2 cm), with a ground electrode placed over the ulnar styloid. Signals were amplified (×1000), band-pass-filtered (10–500 Hz), sampled at 1000 Hz, and recorded using LabChart software (version 8.1.31) with a PowerLab system (ADInstruments, Bella Vista, Australia). Electrode impedance was maintained below 5 kΩ.

A figure-of-eight coil was positioned over the left M1 to induce a posterior–anterior current. The motor hotspot was defined as the location eliciting the largest and most consistent motor evoked potentials (MEPs) in the contralateral FDI muscle. Coil position was marked using scalp landmarks and anatomical references to ensure reproducibility across sessions.

Resting motor threshold (RMT) was defined as the minimum intensity producing MEPs in at least 50% of trials. Single-pulse TMS was delivered at 120% RMT, and 25 MEPs were recorded. Mean peak-to-peak MEP amplitude was used as the index of CSE. Paired-pulse TMS assessed intracortical excitability: SICI: conditioning stimulus at 80% RMT, ISI 3 ms; ICF: conditioning stimulus at 80% RMT, ISI 10 ms; LICI: two suprathreshold pulses, ISI 150 ms. Conditioned MEPs were expressed as a percentage of unconditioned MEPs.

TMS assessments were conducted at baseline, after each treatment block, and at 28-day follow-up where available, in accordance with international safety guidelines.

### 2.6. Data Analysis

Given the exploratory nature of this case series, no inferential statistical analyses were performed. Analyses focused on within-participant descriptive evaluation of longitudinal changes. Clinical outcomes (pain and anxiety) were examined to identify patterns of immediate response, delayed response, rebound during treatment-free intervals, and durability at follow-up. TMS-derived measures (CSE, SICI, LICI, ICF) were interpreted as exploratory neurophysiological indicators and evaluated at the individual level rather than as group-level endpoints. Clinical and neurophysiological trajectories were visually compared within participants; however, no correlation, regression, mediation, or other inferential analysis was conducted to test relationships between these outcome domains. This approach was adopted due to known clinical and neurophysiological heterogeneity in MS and the hypothesis-generating aim of the study.

## 3. Results and Case Descriptions

### 3.1. Cohort Overview

Six participants with MS-related chronic pain were included. Treatment adherence was high: all participants completed at least nine of the ten planned anodal tDCS sessions. No serious adverse events occurred; reported side effects were mild and transient, including scalp tingling, mild headache, and fatigue.

Participants differed substantially in pain phenotype, disease characteristics, baseline symptom burden, and medication exposure (Supplementary Materials). These factors were documented to contextualise individual clinical and TMS-derived trajectories but were not statistically adjusted for in this descriptive case series. Table 1 summarises participant characteristics, intervention completion, and principal response profiles to facilitate comparison across cases.

### 3.2. Overall Clinical and Neurophysiological Patterns

**Figure 2:**
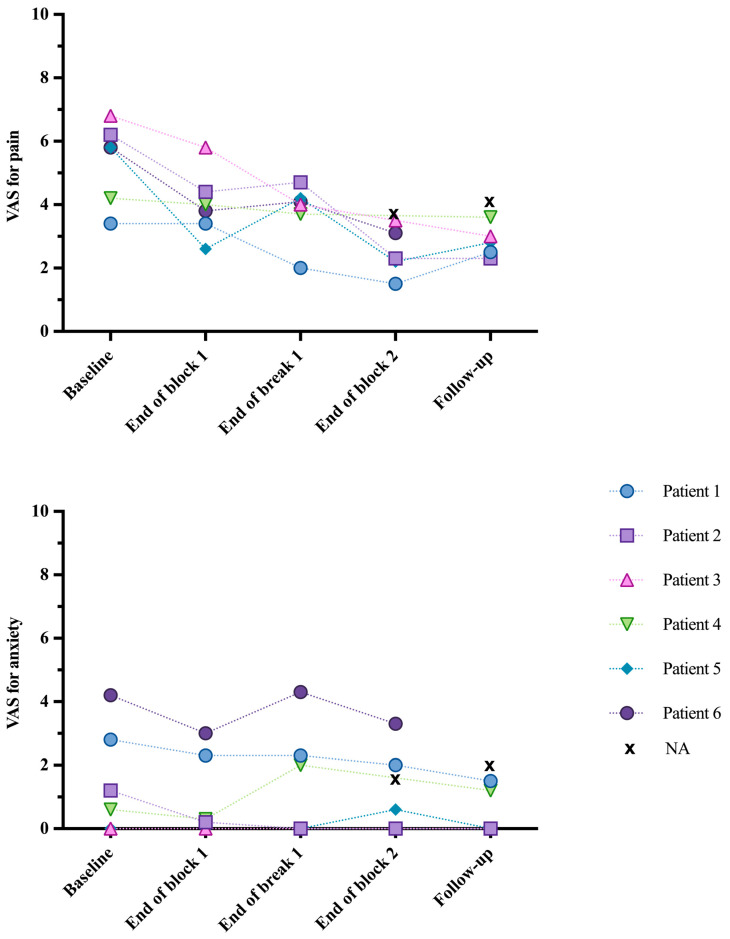
VAS scores for pain and anxiety plotted as a function of time for six individual participants. Visual analogue scale (VAS; 0–10) scores for pain (upper panel) and anxiety (lower panel) are shown at baseline, the end of Block 1, the end of the 2-week inter-block interval, the end of Block 2, and the 4-week follow-up. Each line and symbol represent one participant. Lower scores indicate lower symptom severity. X indicates that data were not available (NA) at the corresponding time point.

Inter-individual heterogeneity was observed across both clinical and TMS-derived measures. Pain, anxiety, and TMS-derived measures differed between participants in the magnitude, timing, direction, and durability of change. Pain reductions were observed in all participants with available post-intervention data (Figure 2), although the extent and persistence of change varied substantially. Four participants demonstrated reductions of ≥40% from baseline to the final available assessment; this threshold was used descriptively and not to define responder status. Anxiety scores also decreased in several participants with elevated baseline anxiety, but this pattern was not consistent across all cases.

**Figure 3:**
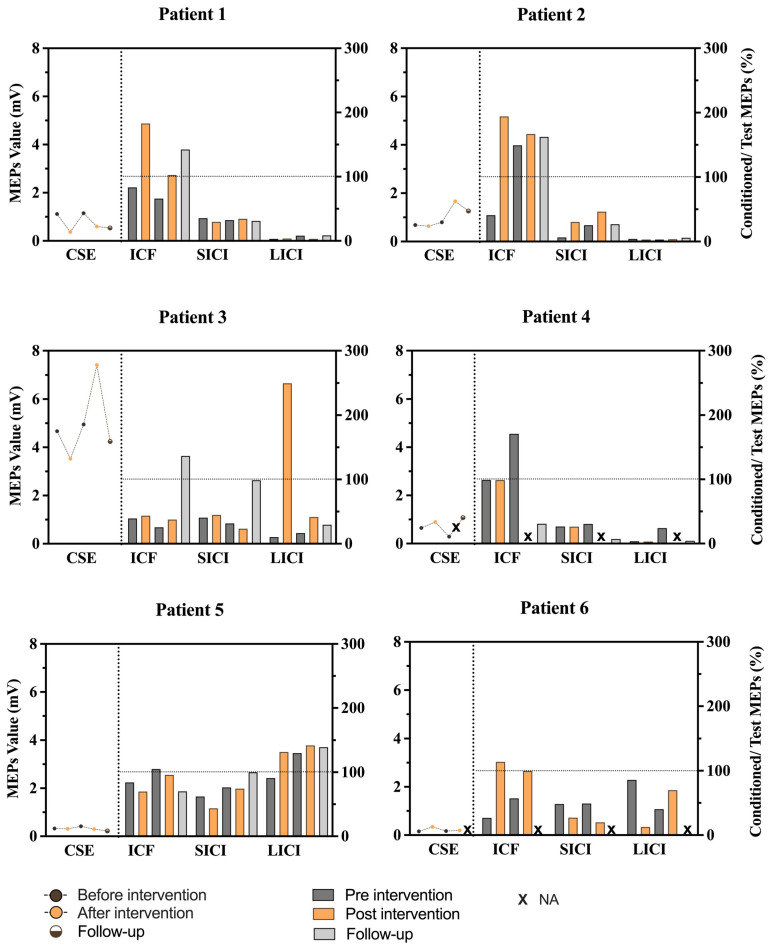
Individual TMS-derived neurophysiological measures across assessment time points. Panels show MEP amplitude as an index of CSE and conditioned/test MEP ratios for ICF, SICI, and LICI. CSE = corticospinal excitability; ICF = intracortical facilitation; SICI = short-interval intracortical inhibition; LICI = long-interval intracortical inhibition; MEP = motor evoked potential; NA = not available.

TMS-derived measures showed similarly heterogeneous patterns (Figure 3). CSE showed no consistent direction of change across participants, and changes in CSE did not uniformly parallel pain reduction. Paired-pulse TMS measures, including ICF, SICI, and LICI, also demonstrated participant-specific changes over time.

### 3.3. Individual Case Descriptions

#### 3.3.1. Case 1

A 42-year-old male with MS-related chronic pain characterised by pins-and-needles sensations and fatigue completed the intervention with one missed session.

Pain remained stable after the first treatment block, decreased by 41.2% at the end of the break, and reached a 55.9% reduction after Block 2. At follow-up, pain remained 26.5% below baseline. Anxiety decreased progressively, with reductions of 17.9% after Block 1 and at the end of the break, 28.6% after Block 2, and 46.4% at follow-up. For TMS-derived measures, CSE showed a marked decrease after Block 1 (−66.7%), returned close to baseline at the end of the break (+3.4%), and was reduced after Block 2 (−46.5%) and at follow-up (−51.9%). ICF showed a large increase after Block 1 (+119.1%), decreased below baseline at the end of the break (−21.2%), and increased again after Block 2 (+23.0%) and follow-up (+70.5%). SICI showed modest reductions across all post-baseline assessments (−15.6% to −2.8%), whereas LICI increased at all post-baseline time points, with the largest increases observed at the end of the break (+175.1%) and follow-up (+199.9%).

Patient perspective: The participant reported that pain relief became noticeable only after completion of the second treatment block and described improved sleep quality at follow-up.

#### 3.3.2. Case 2

A 51-year-old female with severe burning MS-related pain completed all sessions.

Pain decreased by 29.0% after Block 1, showed a smaller 24.2% reduction at the end of the break, and reached a 62.9% reduction after Block 2, which was maintained at follow-up. Anxiety was stable. For TMS-derived measures, CSE changed minimally after Block 1 (−7.1%), increased at the end of the break (+16.5%), and showed larger increases after Block 2 (+143.8%) and at follow-up (+85.0%). ICF was higher than baseline at all post-baseline assessments. SICI also increased across all post-baseline time points, with the largest increase observed after Block 2. LICI decreased during the earlier assessments (−23.1% after Block 1, −27.8% at the end of the break, and −14.8% after Block 2) but increased above baseline at follow-up (+41.6%).

Patient perspective: The participant reported meaningful improvement in daily functioning and reduced sensitivity to pain triggers.

#### 3.3.3. Case 3

A 61-year-old female with severe chronic pain and minimal anxiety completed all sessions.

Pain decreased progressively, with reductions of 14.7% after Block 1, 41.2% at the end of the break, 48.5% after Block 2, and 55.9% at follow-up. Anxiety remained low throughout the observation period. For TMS-derived measures, CSE decreased after Block 1 (−24.4%), increased slightly at the end of the break (+6.0%) and more markedly after Block 2 (+58.8%), before decreasing below baseline at follow-up (−9.1%). ICF showed modest change after Block 1 (+11.0%) and after Block 2 (−4.4%), decreased at the end of the break (−35.0%), and increased substantially at follow-up. SICI showed variable changes over time. LICI showed large percentage increases at all post-baseline assessments.

Patient perspective: The participant reported improved tolerance for daily activities despite persistent MS symptoms.

#### 3.3.4. Case 4

A 53-year-old female with mild allodynia and fatigue completed nine sessions.

Pain showed minimal change, with reductions of 4.8% after Block 1 and 11.9% at the end of the break. Anxiety decreased after Block 1 but increased above baseline at the end of the break and follow-up, indicating an inconsistent anxiety trajectory. For TMS-derived measures, CSE increased after Block 1 (+38.6%), decreased below baseline at the end of the break (−55.5%), and increased above baseline at follow-up (+66.0%); the post-Block 2 assessment was unavailable. ICF remained largely unchanged after Block 1 (−0.5%), increased at the end of the break (+72.2%), and decreased below baseline at follow-up (−68.9%). SICI showed minimal change after Block 1 (−1.0%), increased at the end of the break (+15.0%), and decreased at follow-up (−72.8%). LICI showed variable non-directional changes.

Patient perspective: The participant reported limited perceived benefit but noted mild transient relaxation during stimulation sessions.

#### 3.3.5. Case 5

A 63-year-old female with prominent sensory symptoms completed nine sessions.

Pain showed a rebound–improvement pattern: Pain decreased by 55.2% after Block 1, partially rebounded during the break but remained 27.6% below baseline, and then decreased by 62.1% after Block 2. At follow-up, pain remained 51.7% below baseline. Anxiety remained low throughout the observation period. For TMS-derived measures, CSE showed small decreases after Block 1 (−3.4%) and Block 2 (−7.3%), increased at the end of the break (+30.6%), and decreased at follow-up (−29.7%). ICF decreased after Block 1 (−16.8%), increased at the end of the break (+25.0%) and after Block 2 (+14.1%), and returned below baseline at follow-up (−16.5%). SICI decreased after Block 1 (−29.9%) but increased at the end of the break (+23.3%), after Block 2 (+19.8%), and at follow-up (+60.9%). LICI increased consistently across all post-baseline assessments.

Patient perspective: The participant reported that pain relief was “temporary unless treatment was repeated.”

#### 3.3.6. Case 6

A 50-year-old female with MS-related pain and anxiety completed nine sessions but did not attend follow-up.

Pain and anxiety both decreased following each treatment block, with partial rebound during intervals. Pain decreased by 34.5% after Block 1, remained 29.3% below baseline at the end of the break, and reached a 46.6% reduction after Block 2. Anxiety decreased by 28.6% after Block 1, returned approximately to baseline during the break, and was 21.4% below baseline after Block 2. CSE increased early, followed by partial reduction. ICF increased at all available post-baseline assessments. SICI decreased after Block 1 (−43.7%), returned close to baseline at the end of the break (+1.7%), and decreased again after Block 2 (−59.0%). LICI decreased at all available post-baseline assessments.

Patient perspective: The participant reported improved mood and reduced pain during treatment phases but noted variability related to heat sensitivity and fatigue.

Follow-up status: Missing follow-up data limits the assessment of long-term durability.

### 3.4. Summary of Case Series Findings

In summary, three broad descriptive response profiles were observed across the case series: clinical improvement accompanied by changes in one or more TMS-derived measures; clinical improvement without a corresponding directional change in TMS-derived measures; and minimal or inconsistent changes across clinical and neurophysiological domains. Clinical trajectories included immediate response, delayed response, and rebound–recovery patterns across treatment blocks. TMS-derived measures varied across participants, and the present descriptive data do not allow determination of whether neurophysiological changes were related to clinical outcomes.

## 4. Discussion

This case series describes longitudinal clinical and TMS-derived physiological trajectories following repeated anodal tDCS over M1 in six individuals with MS-related chronic pain. Consistent with CARE reporting principles, the study was designed to characterise within-participant change rather than to estimate treatment efficacy.

The main contribution of this case series is the longitudinal characterisation of response variability. Across participants, repeated anodal tDCS was followed by heterogeneous clinical trajectories and equally heterogeneous TMS-derived changes. Participants differed in the magnitude, timing, and durability of pain change, and clinical trajectories did not consistently align with changes in TMS measures. These descriptive observations suggest that neuromodulation response may be better conceptualised as an individual, time-varying process rather than a fixed treatment effect captured by a single post-intervention endpoint.

### 4.1. Temporal Dynamics of Response to Repeated Stimulation

The observed clinical trajectories are broadly consistent with prior studies showing that repeated anodal tDCS can reduce pain intensity in MS-related chronic pain, with effects persisting beyond the immediate stimulation period [21,24]. However, the two-block longitudinal design used in the present case series provided an opportunity to observe how symptoms changed not only immediately after stimulation, but also during a stimulation-free interval and after re-exposure. In this context, the value of the two-block protocol was not simply additional stimulation exposure, but the ability to separate immediate post-stimulation change, symptom behaviour during a stimulation-free interval, and response to re-exposure. A single pre-post assessment would have obscured several clinically relevant patterns observed here, including delayed improvement, partial rebound during the inter-block interval, and renewed improvement after the second stimulation block.

The rebound–improvement pattern observed in some participants is particularly relevant considering repeated, booster, and maintenance neuromodulation paradigms. Prior neurophysiological work suggests that repeated anodal tDCS delivered within the after-effects of an initial stimulation period can prolong CSE changes, supporting the biological plausibility of interval-based repeated stimulation [23]. More broadly, chronic pain neuromodulation studies and reviews indicate that repeated stimulation sessions are typically required for clinically meaningful benefit, while maintenance or booster strategies may be needed to sustain effects in some conditions [24,25]. In the present case series, rebound during the stimulation-free interval followed by renewed improvement after Block 2 may suggest that some individuals require repeated exposure to maintain benefit. However, this interpretation remains cautious: rebound could also reflect natural symptom fluctuation, regression to the mean, contextual effects, fatigue variation, or other non-specific factors rather than loss of a stimulation-specific effect.

This temporal variability reinforces the value of longitudinal within-participant assessment. In MS-related chronic pain, where symptoms fluctuate and clinical heterogeneity is substantial, response classification based on a single endpoint may be misleading.

### 4.2. CSE and Clinical Variability

CSE, assessed via single-pulse MEPs, demonstrated marked inter-individual variability across participants and time points. Some individuals exhibited increases in CSE, whereas others showed reductions or non-directional fluctuations.

Descriptively, reductions in pain occurred in participants showing increases, decreases, or fluctuations in CSE. Thus, no uniform visual pattern of parallel change between pain and CSE was evident in this small sample. This observation does not establish the absence of a relationship between the two outcomes. This aligns with the known physiological heterogeneity in MS, where motor pathway function may be influenced by lesion burden, fatigue, medication use, and disease duration. This pattern differs from findings reported by Cuypers et al., who observed increased corticospinal output following a single session of anodal tDCS in individuals with MS [26]. However, direct comparison is limited due to differences in study design, stimulation exposure, and outcome focus. Whereas prior work primarily examined acute neurophysiological effects, the present case series evaluated longitudinal pain trajectories across repeated stimulation blocks. Taken together, these observations suggest that changes in CSE may not consistently parallel clinical pain outcomes in MS-related chronic pain.

Accordingly, CSE should be interpreted here as an exploratory physiological descriptor rather than a biomarker of response. Its value in this case series lies in helping to characterise individual physiological trajectories, not in explaining or predicting analgesic response.

### 4.3. Intracortical Circuit Measures and Inter-Individual Variability

Paired-pulse TMS measures (SICI, LICI, and ICF) demonstrated greater inter-individual variability than single-pulse CSE measures, with no consistent directional pattern observed across participants or time points. This variability is consistent with prior neuromodulation literature suggesting that M1 stimulation may influence intracortical inhibitory and facilitatory processes in a heterogeneous manner across individuals [27]. However, interpretation of these measures remains complex, as conditioned motor evoked potentials reflect composite cortical excitability rather than isolated neurotransmitter activity [28]. Therefore, changes in SICI, LICI, or ICF should not be interpreted as evidence that a specific inhibitory or facilitatory pathway mediated clinical change.

The variability observed across these measures may reflect differences in each participant’s physiological starting point. The same stimulation protocol may interact differently with cortical circuits depending on baseline excitability, inhibitory–facilitatory balance, medication exposure, fatigue, disease burden, and network integrity. Variability may not simply represent measurement noise; rather, it may reflect differences in individual susceptibility to stimulation that warrant further investigation [25].

Importantly, the present study was not designed to identify or validate responder subtypes; rather, it highlights the need for future studies using larger samples and multimodal approaches to investigate potential predictors of response.

This interpretation is consistent with emerging precision-neuromodulation frameworks, but the present case series cannot identify predictors of response. Rather, it supports the need for future studies that test whether baseline clinical and physiological profiles can guide stimulation parameters, dosing, timing, or maintenance strategies.

### 4.4. Relationship Between Clinical and Neurophysiological Changes

One of the most important observations was the apparent dissociation between clinical improvement and TMS-derived measures. Because no formal analyses of associations between clinical and neurophysiological outcomes were performed, these observations should be interpreted solely as descriptive patterns rather than evidence of mechanistic relationships. Some participants showed pain reduction without corresponding directional changes in CSE, SICI, LICI, or ICF. These descriptive findings suggest that conventional motor cortical TMS measures may not fully reflect the neurophysiological processes associated with clinical improvement.

This does not mean that motor cortical physiology is irrelevant. M1 stimulation may influence pain through network-level mechanisms that extend beyond corticospinal output, including sensorimotor integration, thalamocortical interactions, descending pain modulation, and affective–cognitive pain networks [29]. These broader processes may not be adequately reflected by MEP amplitude or paired-pulse indices alone.

Thus, the clinical–neurophysiological dissociation observed here is informative. TMS-derived measures may be useful when combined with imaging, sensory testing, and clinical phenotyping to map individual response trajectories.

### 4.5. Affective Symptoms and Symptom Co-Variation

Anxiety trajectories also varied across participants. In some cases, pain and anxiety demonstrated parallel temporal changes, particularly during stimulation-free intervals, whereas in others, pain reduction occurred independently of anxiety changes. These observations indicate that pain and anxiety sometimes changed in parallel, although this pattern was not consistent across all participants. This is consistent with broader literature indicating that psychological distress can influence pain perception and symptom severity in chronic neurological conditions [30]. However, the variability observed here indicates that affective symptoms likely interact with, rather than solely determine, clinical response to neuromodulation.

Clinically, this distinction is important because chronic pain is sometimes misinterpreted as being primarily psychological when anxiety or distress is present. The present observations do not support such a reductionist interpretation. Rather, they suggest that anxiety and pain may co-vary in some individuals, while remaining partially independent in others. Anxiety may amplify pain perception or symptom burden, but it should not be assumed to be the sole cause of pain or the sole explanation for analgesic response. Recognising this distinction may help reduce stigma and unconscious clinician bias and support a multidimensional approach to MS-related chronic pain that validates both neurobiological and affective contributors.

### 4.6. Methodological Considerations

Several methodological limitations should be considered. The absence of a control condition precludes inference regarding treatment efficacy, and observed changes may reflect natural symptom variability, regression to the mean, placebo effects, or non-specific effects of repeated clinical contact. The small sample size and descriptive case-series design also preclude formal testing of relationships between clinical outcomes and TMS-derived measures.

Interpretation of the neurophysiological findings is further limited by the state-sensitive nature of TMS-derived measures. CSE, SICI, LICI, and ICF may be influenced by fatigue, attention, medication use, inter-session variability, and disease-related changes in corticospinal and intracortical function. Participants also differed in MS subtype, disease duration, pain phenotype, baseline symptom burden, and medication exposure, including agents that may influence pain perception, cortical excitability, arousal, fatigue, or affective state. These factors may have contributed to variability in both clinical and TMS-derived trajectories and could not be statistically adjusted for in this case series.

Despite these limitations, the strength of this study lies in its dense longitudinal sampling across repeated stimulation blocks and follow-up. This design allowed observation of clinically relevant response patterns, including immediate improvement, delayed improvement, rebound during stimulation-free intervals, and renewed improvement after re-exposure. These observations are hypothesis-generating and may inform the design of future sham-controlled studies.

Future studies should move beyond single-domain responder definitions. A clinically meaningful response to neuromodulation in MS-related chronic pain may require integrating pain reduction, durability of benefit, affective symptom trajectories, medication context, and multimodal physiological markers. Combining TMS-derived measures with imaging-based lesion metrics, structural and functional connectivity, quantitative sensory testing, and ecological symptom monitoring may help identify response phenotypes rather than simple responder/non-responder categories. Future trials should also examine whether repeated-block, booster, or maintenance stimulation schedules improve the durability of benefit in individuals who show rebound–recovery patterns, using prespecified responder definitions, sham-controlled designs, and longer longitudinal follow-up.

## 5. Conclusions

This case series documents variable longitudinal changes in pain, anxiety, and TMS-derived neurophysiological measures following repeated anodal tDCS over M1 in individuals with MS-related chronic pain. No consistent visual correspondence was observed between the clinical and neurophysiological trajectories. Given the descriptive design and absence of formal analyses of relationships between outcomes, the findings do not establish whether neurophysiological changes contributed to or explained changes in pain or anxiety. These observations are hypothesis-generating and support further controlled studies incorporating prespecified analyses of clinical–neurophysiological relationships.

## Figures and Tables

**Table 1:** Summary of participant characteristics and principal response profiles.

Case	Age/Sex	Main Pain Phenotype	Sessions Completed	Principal Pain Response	Anxiety Response	Exploratory TMS Profile
1	42/M	Pins-and-needles pain; fatigue	9/10	Delayed improvement; greatest reduction after Block 2; partial durability at follow-up	Progressive reduction	Heterogeneous CSE and intracortical changes
2	51/F	Severe burning pain	10/10	Early reduction after Block 1; larger reduction after Block 2; maintained at follow-up	Marked reduction; absent from later assessments	Later CSE increase; intracortical measures variable
3	61/F	Severe chronic pain; minimal anxiety	10/10	Progressive reduction across time points	Absent/minimal throughout	Mixed CSE and intracortical changes
4	53/F	Mild allodynia; fatigue	9/10	Minimal pain change; later pain assessments unavailable	Inconsistent trajectory	Variable non-directional TMS changes
5	63/F	Prominent sensory symptoms	9/10	Rebound–improvement pattern; benefit after repeated stimulation	Not clearly emphasized/variable	Stable CSE; variable ICF and higher follow-up SICI and LICI ratios
6	50/F	MS-related pain with anxiety	9/10	Block-related improvement with partial rebound; follow-up unavailable	Block-related reduction with rebound	Heterogeneous changes; follow-up missing

Note. CSE = corticospinal excitability; F = female; ICF = intracortical facilitation; LICI = long-interval intracortical inhibition; M = male; MS = multiple sclerosis; SICI = short-interval intracortical inhibition; TMS = transcranial magnetic stimulation. TMS-derived measures are summarized descriptively and should be interpreted as exploratory physiological descriptors rather than biomarkers of response.

## Data Availability

The data are not publicly available due to privacy and ethical restrictions, as they contain potentially identifiable patient information from a small clinical case series. Data may be available from the corresponding author upon reasonable request and subject to ethics approval.

## Supplementary Materials

The following supporting information can be downloaded at: https://www.mdpi.com/article/10.3390/brainsci16070767/s1. Table S1: Characteristics of participants with multiple sclerosis and chronic pain.
