# Supplementary material for: Long-Lasting Changes of Repeated Anodal Transcranial Direct Current Stimulation in Decreasing Chronic Pain in Patients with Multiple Sclerosis: A Case Series
Source: Brain Sci. 2026 Jul 22;16(7):767. doi: 10.3390/brainsci16070767 (PMC13407326; doi:10.3390/brainsci16070767)
Supplement: Supplementary file 1 [file brainsci-16-00767-s001.zip › brainsci-4405974-supplementary.pdf]

Table S1: Characteristics of participants with multiple sclerosis and chronic pain.

Case	Age and sex	MS subtype	Disease/pain duration	Medication use	Pain site	Pain characteristics
1	42, M	PPMS	2/2 years	Brintellix 10mg daily<br>Quetiapine 50 mg daily<br>Baclofen 10mg daily<br>Clonazepam 0.5mg daily<br>Ezetimibe 10mg daily<br>Nexium 20mg daily	Lower leg and back pain	1. SF-MPQ-2<br>(1) Total score: 75/220<br>(2) Mean score: 3.4/10<br>(3) Main pain type: neuropathic pain<br>(4) PPI: 3<br>(5) Overall: discomforting<br>2. NPSI<br>(1) Total score: 38/100<br>(2) Dominant NPSI symptoms: paresthesia/dysesthesia<br>(3) Temporal pattern: spontaneous pain permanently present; 6–10 attacks/24 hours
2	51, F	RRMS	29/20 years	Baclofen 10 mg every 4 hours<br>Esomeprazole 40mg daily<br>Irbesartan 75mg daily<br>Pregabalin 300mg twice daily<br>Ivabradine 5mg twice daily<br>Rosuvastin 20mg daily<br>Picrolimus cream<br>Clopidogrel 75mg daily	Widespread pain affecting four limbs and back sparing abdomen and chest	1. SF-MPQ-2<br>(1) Total score: 98/220<br>(2) Mean score: 4.45/10<br>(3) Main pain type: continuous pain<br>(4) PPI: 0<br>(5) Overall: no pain<br>2. NPSI<br>(1) Total score: 19/100<br>(2) Dominant NPSI symptoms: burning pain and electric-shock-like pain

						(3) Temporal pattern: persistent spontaneous pain; >20 attacks/24 hours
3	61, F	RRMS	18/12 years	Oxazepam15 mg daily<br>Targin 2.5/1.25 daily<br>Baclofen 10mg daily<br>Gabapentin 100mg daily<br>Sevikar 40/5/25mg daily<br>Propranolol 40mg daily	Neck, bilateral hands, and bilateral lower legs below the knees	1. SF-MPQ-2<br>(1) Total score: 93/220<br>(2) Mean score: 4.23/10<br>(3) Main pain type: Intermittent and neuropathic pain<br>(4) PPI: 0<br>(5) Overall: no pain<br>2. NPSI<br>(1) Total score: 58/100<br>(2) Dominant NPSI symptoms: paroxysmal pain and paresthesia/dysesthesia<br>(3) Temporal pattern: spontaneous pain present 8–12 hours/day; 6–10 attacks/24 hours
4	53, F	RRMS	18/15 years	Duloxetine 60mg daily<br>Clonidine 100-200mcg daily<br>Gabapentin 100mg daily<br>LGP THC 7.5mg daily	Widespread pain involving the scalp, bilateral shoulders and upper arms, thoracic and lumbar spine, with posterior radiation to both legs; sparing the forearms, chest, abdomen, and anterior	1. SF-MPQ-2<br>(1) Total score: 66/220<br>(2) Mean score: 3/10<br>(3) Main pain type: neuropathic pain<br>(4) PPI: 5<br>(5) Overall: Discomforting<br>2. NPSI<br>(1) Total score: 44/100<br>(2) Dominant NPSI symptoms: evoked

					aspects of both legs	pain, with prominent spontaneous pain<br>(3) Temporal pattern: spontaneous pain permanently present; 1–5 attacks/24 hours
5	63, F	RRMS	21/15 years	Amitriptyline<br>Ezetimbe/Simvastatin<br>Irebesatan<br>Siponimod (Mayzent)<br>Moduretic<br>Nexium<br>Nizac<br>Ovestin HRT<br>Pregabalin	Lower body pain involving the right flank/abdominal region around the T10 intercostal band, the entire right leg, and the left leg below the knee	1. SF-MPQ-2<br>(1) Total score: 36/220<br>(2) Mean score: 1.64/10<br>(3) Main pain type: neuropathic pain<br>(4) PPI: 0<br>(5) Overall: no pain<br>2. NPSI<br>(1) Total score: 64/100<br>(2) Dominant NPSI symptoms: paresthesia/dysesthesia and spontaneous pain<br>(3) Temporal pattern: spontaneous pain permanently present; 1–5 attacks/24 hours
6	50, F	RRMS, inactive	15/10 years	Zopiclone 7.5mg nocte<br>Nitrofurantoin<br>Escitalopram 40mg daily<br>Esomeprazole 20mg bd<br>Mounjaro<br>Periodic ABs for UTIs	Bilateral forearms below the elbows, bilateral lower legs below the knees, and bilateral buttocks	1. SF-MPQ-2<br>(1) Total score: 77/220<br>(2) Mean score: 3.5/10<br>(3) Main pain type: neuropathic pain<br>(4) PPI: 7<br>(5) Overall: discomforting<br>2. NPSI<br>(1) Total score: 40/100

	(2) Dominant NPSI symptoms: paresthesia/dysesthesia, with spontaneous and paroxysmal pain<br>(3) Temporal pattern: Spontaneous pain permanently present; >20 attacks/24 hours

Abbreviations: F, female; HRT, hormone replacement therapy; LGP THC, Little Green Pharma tetrahydrocannabinol; M, male; mcg, microgram; mg, milligram; MS, multiple sclerosis; NPSI, Neuropathic Pain Symptom Inventory; PPMS, primary progressive multiple sclerosis; PPI, Present Pain Intensity; RRMS, relapsing-remitting multiple sclerosis; SF-MPQ-2, Short-Form McGill Pain Questionnaire-2; T10, tenth thoracic dermatome/intercostal level; UTIs, urinary tract infections.
